# Heritability and Preliminary Genome-Wide Linkage Analysis of Arsenic Metabolites in Urine

**DOI:** 10.1289/ehp.1205305

**Published:** 2013-01-15

**Authors:** Maria Tellez-Plaza, Matthew O. Gribble, V. Saroja Voruganti, Kevin A. Francesconi, Walter Goessler, Jason G. Umans, Ellen K. Silbergeld, Eliseo Guallar, Nora Franceschini, Kari E. North, Wen H. Kao, Jean W. MacCluer, Shelley A. Cole, Ana Navas-Acien

**Affiliations:** 1 Department of Epidemiology, and; 2 Department of Environmental Health Sciences, Johns Hopkins Bloomberg School of Public Health, Baltimore, Maryland, USA; 3 Area of Epidemiology and Population Genetics, National Center for Cardiovascular Research (CNIC), Madrid, Spain; 4 Fundacion de Investigacion del Hospital Clinico de Valencia-INCLIVA, Valencia, Spain; 5 Department of Genetics, Texas Biomedical Research Institute, San Antonio, Texas, USA; 6 Institute of Chemistry–Analytical Chemistry, Karl-Franzens University, Graz, Austria; 7 MedStar Health Research Institute, Hyattsville, Maryland, USA; 8 Georgetown–Howard Universities Center for Clinical and Translational Science, Washington, DC, USA; 9 Welch Center for Prevention, Epidemiology and Clinical Research, and; 10 Department of Medicine, Johns Hopkins Medical Institutions, Baltimore, Maryland, USA; 11 Department of Epidemiology, School of Public Health, University of North Carolina at Chapel Hill, Chapel Hill, North Carolina, USA

**Keywords:** American Indians, arsenic metabolism, arsenic species, determinants, heritability, linkage scan, Strong Heart Study

## Abstract

Background: Arsenic (III) methyltransferase *(AS3MT)* has been related to urine arsenic metabolites in association studies. Other genes might also play roles in arsenic metabolism and excretion.

Objective: We evaluated genetic determinants of urine arsenic metabolites in American Indian adults from the Strong Heart Study (SHS).

Methods: We evaluated heritability of urine arsenic metabolites [percent inorganic arsenic (%iAs), percent monomethylarsonate (%MMA), and percent dimethylarsinate (%DMA)] in 2,907 SHS participants with urine arsenic measurements and at least one relative within the cohort. We conducted a preliminary linkage analysis in a subset of 487 participants with available genotypes on approximately 400 short tandem repeat markers using a general pedigree variance component approach for localizing quantitative trait loci (QTL).

Results: The medians (interquartile ranges) for %iAs, %MMA, and %DMA were 7.7% (5.4–10.7%), 13.6% (10.5–17.1%), and 78.4% (72.5–83.1%), respectively. The estimated heritability was 53% for %iAs, 50% for %MMA, and 59% for %DMA. After adjustment for sex, age, smoking, body mass index, alcohol consumption, region, and total urine arsenic concentrations, LOD [logarithm (to the base of 10) of the odds] scores indicated suggestive evidence for genetic linkage with QTLs influencing urine arsenic metabolites on chromosomes 5 (LOD = 2.03 for %iAs), 9 (LOD = 2.05 for %iAs and 2.10 for %MMA), and 11 (LOD = 1.94 for %iAs). A peak for %DMA on chromosome 10 within 2 Mb of *AS3MT* had an LOD of 1.80.

Conclusions: This population-based family study in American Indian communities supports a genetic contribution to variation in the distribution of arsenic metabolites in urine and, potentially, the involvement of genes other than *AS3MT*.

Exposure to inorganic arsenic (iAs) from water, food, and ambient air is widespread (Franklin et al. 2008; Gilbert-Diamond et al. 2011; Nordstrom 2002). Humans metabolize iAs (arsenate and arsenite) to methylated compounds [predominantly monomethylarsonate (MMA) and dimethylarsinate (DMA)], which are largely excreted in urine together with iAs (Cullen and Reimer 1989; Naranmandura et al. 2006; Vahter 2002). In human populations, the average distribution of arsenic metabolites in urine is approximately 10–30% iAs, approximately 10–20% MMA, and approximately 60–80% DMA (Chiou et al. 1997; Gamble et al. 2005; Gomez-Rubio et al. 2012; Hopenhayn-Rich et al. 1996b; Vahter 2000). However, substantial interindividual variation is found in the distribution of urine arsenic metabolites. Understanding the determinants of arsenic metabolism is important because differences in arsenic methylation patterns in urine have been associated with differential risks of skin lesions, cancer, and cardiovascular disease in several populations exposed to arsenic in drinking water (Chen et al. 2003; Del Razo et al. 1997; Hsueh et al. 1997; Kile et al. 2011; Steinmaus et al. 2006; Wu et al. 2006; Yu et al. 2000).

Polymorphisms in arsenic (III) methyltransferase (*AS3MT*) have been consistently associated with urine arsenic methylation patterns in populations from Argentina (Schlawicke et al. 2007, 2009), Chile (Hernandez et al. 2008), Mexico (Gomez-Rubio et al. 2010; Meza et al. 2005), and Central Europe (Lindberg et al. 2007). Variation near the *AS3MT* gene has also been recently associated with arsenic metabolism in a genome-wide association study from Bangladesh (Pierce et al. 2012). Functional studies have confirmed the relevance of *AS3MT* in the methylation of arsenic (Chen et al. 2011; Drobna et al. 2006; Thomas et al. 2004; Wood et al. 2006). Those studies, however, also suggest that other genes, in addition to nongenetic factors, may contribute to arsenic methylation and distribution in human tissues, although the genes involved remain largely unknown. Genome-wide genetic approaches may contribute to the discovery of genes related to variation in urine arsenic metabolites. Moreover, while arsenic metabolism shows evidence for familial aggregation (Chung et al. 2002), the heritability of urine arsenic methylation patterns has not been evaluated.

The Strong Heart Study (SHS) is a population-based prospective cohort study funded by the National Heart, Lung, and Blood Institute (NHLBI) to evaluate cardiovascular disease and its risk factors, including genetic and environmental determinants, in 13 U.S. American Indian communities from Arizona, Oklahoma, and North and South Dakota (Lee et al. 1990). Some of these communities are known to be exposed to arsenic in drinking water (Navas-Acien et al. 2009). In this study, we first evaluated the heritability of urine arsenic methylation patterns in SHS participants who had at least one relative within the cohort. In a subset of the population with genome-wide short tandem repeat (STR) markers available, we conducted a preliminary study to evaluate the presence of genetic loci associated with the distribution of urine arsenic metabolites by conducting a genome-wide quantitative trait locus (QTL) linkage scan.

## Methods

*Study population.* From 1989 to 1991, all men and women 45–74 years of age from selected communities in Arizona and Oklahoma were invited to participate in the SHS (Lee et al. 1990). In North and South Dakota, a cluster sampling technique was used. Of all the individuals invited, 62% agreed to participate. Participants were similar to nonparticipants in age, body mass index (BMI), and self-reported frequency of diabetes. Women were more likely to participate than men. Starting in 1998, the Strong Heart Family Study (SHFS) recruited persons who were ≥ 18 years of age and extended family members of the original SHS participants to participate in a study of the genes that contribute to cardiometabolic risk in American Indian populations (North et al. 2003). For the SHFS, families who had at least five living siblings, including three original SHS participants, were invited; and parents, spouses, offspring, spouses of offspring, and grandchildren were enrolled to build extended pedigrees. The SHFS genotyped genome-wide STR markers in all participants.

Urine metals, including the arsenic species inorganic arsenic, MMA and DMA, were measured in 3,974 individual persons who participated in the SHS baseline visit (1989–1991) (Scheer et al. 2012). For the present analysis, we excluded 1 participant who was missing total arsenic, and 1 participant missing inorganic arsenic concentrations. We further excluded 222 participants whose %iAs, %MMA, or %DMA were below the limit of detection. We also excluded 5 participants who were missing information on smoking, 9 participants missing information on alcohol consumption, 16 participants missing BMI measurement, 1 participant missing level-of-education information, and 5 participants missing urine creatinine, leaving a sample size of 3,714 SHS participants. Among those, 2,907 SHS participants had at least 1 relative within the cohort, allowing heritability analysis and 487 were also SHFS participants with STR marker information for the linkage analysis.

The 13 participating tribes, the Indian Health Service (IHS) Institutional Review Board (IRB), and the IRBs of the participating institutions approved the SHS and SHFS protocol and consent forms. All participants provided written and oral informed consent at enrollment into the SHS. The present heritability and linkage study was covered by the original SHS consent form because arsenic is a potential cardiovascular risk factor. In addition, the present study was specifically approved by the SHS Publications and Presentations Committee and by the participating tribes.

*Urine arsenic*. Spot urine samples were collected in 1989–1991, frozen within 1–2 hr of collection, and stored at –80ºC at the Penn Medical Laboratory, MedStar Health Research Institute (Hyattsville, MD, and Washington, DC, USA) (Lee et al. 1990). In 2009, up to 1.0 mL of urine from each participant was transported on dry ice to the Trace Element Laboratory of the Institute of Chemistry–Analytical Chemistry, Karl-Franzens University (Graz, Austria). There, total arsenic concentrations in the urine samples were measured by inductively coupled plasma mass spectrometry (ICPMS) (Agilent model 7700× ICPMS; Agilent Technologies, Waldbronn, Germany), and arsenic species were determined by high performance liquid chromatography (HPLC; Agilent model 1100) coupled to ICPMS and served as the arsenic selective detector (HPLC/ICPMS). The analytical methods used to determine urine arsenic concentrations have been described in detail (Scheer et al. 2012). The limits of detection were 0.2 µg/L for total arsenic, and 0.1 µg/L for iAs, MMA, DMA, and for arsenobetaine plus other cations. Participants with iAs, MMA, and DMA below the limits of detection (5.3% for iAs, 0.7% for MMA, 0.03% for DMA) were excluded from this analysis because it is not possible to evaluate arsenic metabolism with undetectable urine arsenic biomarkers. An in-house reference urine and the NIES No. 18 Human Urine from the National Institute for Environmental Studies (Ibaraki, Japan) were analyzed together with the samples. The interassay coefficients of variation for the in-house reference urine for total arsenic, iAs, MMA, DMA, and arsenobetaine plus other cations were 4.4%, 6.0%, 6.5%, 5.9%, and 6.5%, respectively.

Urine arsenic data were transmitted to the Texas Biomedical Research Institute (previously known as Southwest Foundation for Biomedical Research) where they were transferred to the pedigree data management system PEDSYS (Dyke 1999).

*Demographic and lifestyle assessment*. Baseline sociodemographic, lifestyle, and anthropometric information was obtained through interview and physical examination conducted by trained nurses and medical assistants (Lee et al. 1990). The standardized in-person questionnaire included sociodemographic data (age, sex, education) and smoking status (never, current, former). Body mass index (BMI) was estimated by dividing measured weight in kilograms by measured height in meters squared.

*Short tandem repeat markers*. DNA from white cells was isolated and stored at the Texas Biomedical Research Institute. We genotyped nearly 400 STR markers, spaced, on average 10 cM (centimorgans) apart (range, 2.4–24.1 cM) [For details, see Supplemental Material, Table S3 (http://dx.doi.org/10.1289/ehp.1205305)], using ABI PRISM Linkage Mapping Set-MD10 version 2.5 (Applied Biosystems, Foster City, CA, USA). Individual polymerase chain reaction (PCR) products were loaded into an ABI PRISM 377 Genetic Analyzer for laser-based automated genotyping. Genotypes were assigned using the Genotyper software system (Applied Biosystems). Pedigree relationships were verified and likely genotyping errors were detected using PREST software version 3.02 (Pedigree Relationship Statistical Tests, http://utstat.toronto.edu/sun/Software/Prest/prest3.02/) (McPeek and Sun 2000; Sun et al. 2002) and SimWalk2 (Sobel et al. 2002). Mendelian inconsistencies and unlikely double recombinants in marker genotypes were removed with an overall blanking rate of < 1% in the total study population. The average heterozygosity was 0.69 for Arizona, 0.74 for Oklahoma, and 0.76 for North and South Dakota.

*Statistical analysis*. Descriptive analysis. To evaluate urine arsenic methylation and excretion patterns, we computed the proportions of iAs, MMA, and DMA by dividing the concentration of each species by the sum of all three species and multiplying by 100, yielding %iAs, %MMA, and %DMA. The median [interquartile range (IQR)] percentages of urine arsenic species were reported for the overall population and according to age (≤ 55 years, > 55 years), sex (men, women), education (< 12 years completed, ≥ 12 years completed), BMI (< 30 kg/m^2^, ≥ 30 kg/m^2^), smoking (never, former, current), alcohol consumption (never, former, current), and study region (Arizona, Oklahoma, and North and South Dakota).

Heritability. We estimated the heritability of %iAs, %MMA, and %DMA using a general pedigree variance components decomposition-based method as conducted by the software Sequential Oligogenic Linkage Analysis Routines (SOLAR) version 4.4.0 (Almasy et al. 2012). For a detailed description of the statistical methods used to estimate heritability, see Supplemental Material, pp. 2–3 (http://dx.doi.org/10.1289/ehp.1205305). In brief, SOLAR incorporates the information contained in participant pedigrees to obtain maximum likelihood estimates for the proportion of unexplained variance due to additive genetic effects from polygenes (σ^2^_g_) and the proportion of variance due to unmeasured environmental covariates, measurement error, and nonadditive genetic effects (σ^2^_e_). Heritability (h^2^) is defined as the proportion of unexplained variance in the observed distribution of the percent of each urine arsenic species that is attributable to additive genetic effects, or h^2^ = σ^2^_g_/(σ^2^_g_ + σ^2^_e_). The *p*-values for h^2^ are computed from a likelihood ratio test comparing the model in which the h^2^ component of the unexplained variance is estimated to a model in which h^2^ is constrained to be zero, following a 1/2:1/2 mixture of chi-square distributions with 1 degree of freedom (df) and a point mass at zero (Amos 1994).

Initially, the percentage of each urine arsenic species was converted to an odds and introduced in a linear variance component model as a logit-transformed dependent variable adjusted for age, sex, age^2^, age × sex and age^2^ × sex, education (< 12 years of education, ≥ 12 years of education), BMI (< 30 kg/m^2^, ≥ 30 kg/m^2^), smoking status (never, former, current smokers), alcohol drinking status (never, former, current drinkers), region (Arizona, Oklahoma, and North and South Dakota), and total urine arsenic concentrations. To reduce kurtosis, residuals from the linear variance component model were transformed using an inverse Gaussian transformation and introduced as dependent variables in a second-stage variance component linear regression with no covariables. A household component of the variance was explored to account for “shared environment” among relatives living in the same household, but household component was not retained in the final model because it did not influence the heritability estimates (data not shown). We performed heritability analysis for the population as a whole, and stratified by region.

Linkage scan. We implemented a multipoint QTL linkage analysis based on variance components decomposition-based methods in SOLAR version 4.4.0 (Almasy and Blangero 1998; Almasy et al. 2012), building on the heritability models described above by including additional variance components for QTLs based on multipoint identity-by-descent (IBD) matrices [for details on model formulation, see Supplemental Material, pp, 2–3 (http://dx.doi.org/10.1289/ehp.1205305)]. We computed multipoint IBD matrices using the software Loki (Heath 1997; Heath et al. 1997) by using STR marker map positions obtained based on the DeCode map (NHLBI 2012). At each chromosomal location, SOLAR conducts likelihood ratio tests comparing a model that estimates the unexplained variance attributable to a potential QTL versus a model that constrains the unexplained variance attributable to the potential QTL to be equal to zero. The tests at each chromosomal location are reported as LOD [logarithm (to the base of 10) of the odds] scores in favor of genetic linkage with a QTL. LOD scores of 1.9 and 3.3 are considered suggestive and significant evidence, respectively, for a QTL (Lander and Kruglyak 1995). To evaluate whether the linkage findings may have been due to chance, we conducted an adjustment analysis in order to calculate an empirical LOD score for each urine arsenic metabolite. The empirical LOD scores were computed by multiplying the original LOD scores with a correction constant. The correction constant is calculated when a fully informative marker, not associated with the phenotype, is simulated and goes through approximately 10,000 replicates, IBDs are calculated for this marker and the LOD score is computed for linkage of this phenotype to this marker. In this analysis, if the original LOD score is similar to the adjusted LOD score, then the possibility of findings being due to chance alone is low.

*Sensitivity analyses.* Several sensitivity analyses were conducted for both the heritability and the linkage scan. First, we adjusted for BMI as continuous rather than categorical. Second, we added an interaction term for BMI (categorical) × sex. Third, we further adjusted for urine selenium. Fourth, we accounted for urine dilution by adjusting for urine creatinine rather than by dividing by urine creatinine. Last, we repeated the analyses without accounting for urine dilution. The heritability and linkage scan analyses remained unchanged (data not shown).

## Results

*Descriptive analysis*. Median (IQR) total urine arsenic concentrations were 18.5 (12.5–27.2) µg/g in Arizona, 8.2 (5.6–12.9) µg/g in Oklahoma, and 12.5 (8.2–18.9) µg/g in North and South Dakota (Table 1). The median (IQR) urine arsenobetaine concentration was 0.7 (0.4–1.6) µg/g, consistent with the low self-reported seafood consumption in the study population. The median (IQR) for %iAs, %MMA, and %DMA were 7.7 (5.4–10.7)%, 13.6 (10.5–17.1)%, and 78.4 (72.5–83.1)%, respectively, with no differences by total urine arsenic concentrations above and below the median (Table 2). The %MMA was higher in men compared with women, in participants from North and South Dakota compared with other regions, in participants with BMI < 30 kg/m^2^ compared with ≥ 30 kg/m^2^, and current smokers compared with nonsmokers (Table 2). Urine arsenic levels and participant characteristics in the subsample of 487 participants with information for the linkage analysis were similar to those of the larger study sample included in the heritability analysis [see Supplemental Material, Tables S1 and S2 (http://dx.doi.org/10.1289/ehp.1205305)]. Total urine arsenic concentrations in the full study sample were weakly correlated with %iAs and %MMA, but not correlated with %DMA (see Supplemental Material, Figure S1). The %iAs and %MMA were moderately positively correlated (Spearman correlation coefficient 0.46). The %DMA was strongly negatively correlated with %iAs and %MMA (Spearman correlation coefficients –0.81 and –0.87, respectively).

**Table 1:** Characteristics of SHS participants having at least one relative within the cohort.

	Arizona	Oklahoma	North/South Dakota	Overall
Total (n)	1,259	1,139	509	2,907
Age (years)	55.4 ± 0.2	56.6 ± 0.2	56.0 ± 0.4	56.0 ± 0.1
Sex (% males)	36.2 (1.4)	43.7 (1.5)	43.8 (2.2)	40.5 (0.9)
Education (% < high school)	60.8 (1.4)	30.5 (1.4)	47.9 (2.2)	46.6 (0.9)
BMI (kg/m2)	36.5 ± 0.2	30.7 ± 0.2	29.9 ± 0.3	31.4 ± 0.1
Smoking status (%)
Former	35.1 (1.4)	36.1 (1.4)	30.5 (2.0)	35.3 (0.9)
Current	19.5 (1.1)	35.1 (1.4)	46.6 (2.2)	30.4 (0.9)
Current alcohol drinkers (%)
Former	39.6 (1.4)	45.4 (1.5)	38.2 (2.2)	41.7 (0.9)
Current	41.9 (1.4)	37.9 (1.4)	50.9 (2.2)	41.9 (0.9)
Total arsenic [median (IQR), μg/g]	18.5 (12.5–27.2)	8.2 (5.6–12.9)	12.5 (8.2–18.9)	12.9 (7.9–21.0)
Arsenobetaine [median (IQR), μg/g]	0.8 (0.5–1.7)	0.7 (0.4–1.8)	0.5 (0.3–1.0)	0.7 (0.4–1.6)
Urine selenium [median (IQR), μg/g]	48.0 (36.5–64.3)	42.2 (33.9–55.4)	63.7 (47.4–85.3)	47.7 (36.1–64.9)
Data are presented as percentage (SE) for categorical variables or mean ± SE for continuous variables.				

**Table 2:** Median (IQR) of percentage urine arsenic species in the SHS participants having at least one relative within the cohort.

	n	%iAs	%MMA	%DMA
Overall	2,907	7.7 (5.4–10.7)	13.6 (10.5–17.1)	78.4 (72.5–83.1)
Age (years)
≤ 55	1,481	8.3 (5.8–11.2)	13.6 (10.4–17.2)	77.8 (72.1–82.8)
> 55	1,426	7.2 (5.1–10.1)	13.6 (10.6–17.0)	79.1 (73.0–83.5)
Sex
Men	1,177	9.2 (6.4–12.7)	15.4 (12.1–18.9)	75.0 (68.8–80.7)
Women	1,730	6.9 (4.9–9.4)	12.6 (9.6–15.7)	80.2 (75.3–84.4)
Study region
Arizona	1,259	8.6 (6.1–11.5)	13.3 (10.4–16.4)	78.0 (72.6–82.5)
Oklahoma	1,139	6.6 (4.6–9.3)	13.3 (10.2–16.8)	79.6 (74.0–84.3)
North and South Dakota	509	8.1 (5.9–11.5)	15.3 (11.9–19.4)	76.5 (69.2–81.6)
Education (years)a
< 12	1,551	7.4 (5.3–10.6)	13.5 (10.3–16.9)	78.7 (72.7–83.5)
≥ 12	1,356	8.1 (5.7–11.0)	13.6 (10.8–17.3)	78.2 (72.2–82.6)
BMI (kg/m2)b
< 30	1,329	8.3 (5.7–11.4)	15.2 (11.8–18.6)	76.4 (70.2–81.3)
≥ 30	1,578	7.3 (5.3–10.1)	12.5 (9.7–15.6)	79.9 (74.5–84.2)
Smoking
Never	998	7.3 (5.3–10.0)	13.2 (10.0–16.0)	79.4 (74.2–83.7)
Former	1,026	7.6 (5.3–10.5)	13.3 (10.4–16.9)	78.8 (72.9–83.7)
Current	883	8.6 (5.8–11.8)	14.8 (11.2–18.6)	76.6 (70.1–81.8)
Alcohol drinking
Never	476	7.1 (5.2–9.5)	13.4 (10.5–16.3)	79.6 (74.0–83.4)
Former	1,212	7.5 (5.3–10.7)	13.6 (10.5–17.0)	78.4 (72.6–83.5)
Current	1,219	8.2 (5.7–11.2)	13.7 (10.5–17.5)	77.8 (71.7–82.8)
Total arsenic (µg/g)c
< 12.9	1,452	7.3 (5.2–10.1)	14.3 (11.2–17.8)	78.2 (72.3–82.7)
≥ 12.9	1,455	8.2 (5.8–11.4)	13.0 (9.9–16.4)	78.8 (72.7–83.5)
Arsenobetaine (µg/g)c
< 0.7	1,448	8.0 (5.7–10.9)	14.3 (11.0–17.6)	77.6 (71.9–82.2)
≥ 0.7	1,459	7.5 (5.1–10.5)	13.1 (10.0–16.5)	79.4 (73.5–83.9)
Urine selenium (µg/g)c
< 47.7	1,452	7.8 (5.4–11.0)	14.2 (10.9–17.7)	77.7 (71.6–82.6)
≥ 47.7	1,455	7.7 (5.5–10.5)	13.1 (10.1–16.3)	79.1 (73.5–83.5)
aFor education we selected a cut-off of 12 years, consistent with the completion of high school. bFor BMI we selected a cut-off of 30 kg/m2, commonly used to classify individuals as obese and nonobese. cThe cut-off is the median.				

*Heritability*. After accounting for age, sex, education, smoking, alcohol consumption, BMI, and region, the estimated residual heritability was 53% for %iAs, 50% for %MMA, and 59% for %DMA, with similar estimates when stratified by study region (Table 3).

**Table 3:** Heritability of urine arsenic metabolites in SHS participants having at least one relative within the original cohort.

	Heritability (SE)	p-Value	Percent variance explained by covariables
Overall (n = 2,907)
%iAs	0.53 (0.07)	< 0.001	15.5%
%MMA	0.50 (0.07)	< 0.001	19.4%
%DMA	0.59 (0.06)	< 0.001	20.4%
Arizona (n = 1,259)
%iAs	0.46 (0.12)	< 0.001	11.8%
%MMA	0.51 (0.12)	< 0.001	15.1%
%DMA	0.60 (0.11)	< 0.001	16.4%
Oklahoma (n = 1,139)
%iAs	0.50 (0.12)	< 0.001	10.6%
% MA	0.53 (0.11)	< 0.001	22.6%
%DMA	0.62 (0.10)	< 0.001	20.9%
North and South Dakota (n = 509)
%iAs	0.60 (0.11)	< 0.001	19.6%
%MMA	0.36 (0.12)	0.001	19.9%
%DMA	0.55 (0.11)	< 0.001	23.4%
Adjusted for age, age2, sex, age × sex, age2 × sex, smoking status (never, former, current), education (< 12 years, ≥12 years), BMI (< 30 kg/m2, ≥ 30 kg/m2), alcohol status (never, former, current), location (Arizona, Oklahoma, North and South Dakota), and total arsenic (log µg/g). Residual kurtosis ranged from –0.14 to –0.04. The number of pair relationships in the overall sample is distributed as follows: 93 parent–offspring; 1,142 siblings; 271 avuncular; 73 half siblings; 3 double first cousins; 4 grand avuncular; 32 half avuncular; 172 first cousins; 9 half first cousins and half second cousins; 13 first cousins once removed (1 rem); 9 half first cousins; 9 half first cousins, 1 rem, and half second cousins, 1 rem.			

**Figure 1:**
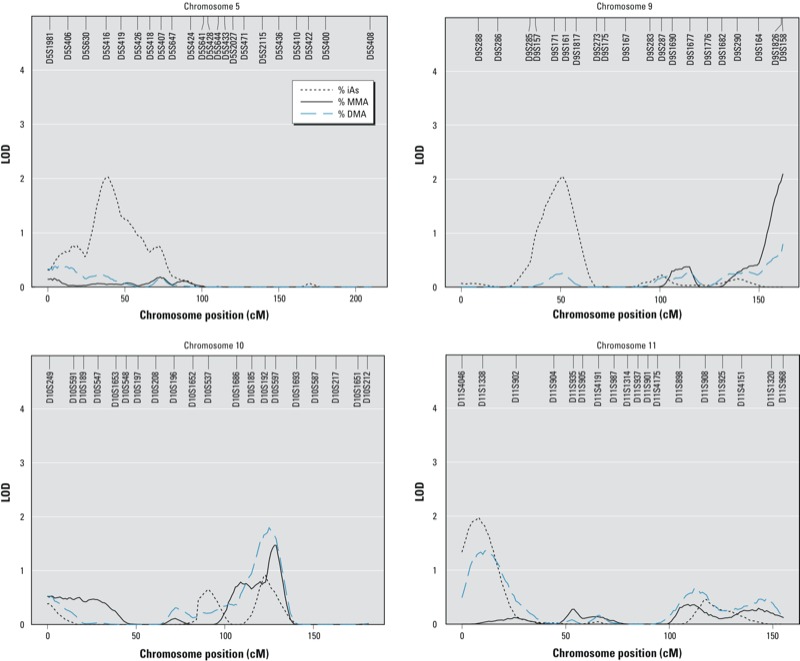
LOD scores on chromosomes 5, 9, 10, and 11 for urine arsenic metabolites in SHS participants with STR marker genotype (*n* = 487). Models were adjusted for age, age^2^, sex, age × sex, age^2^ × sex, smoking status (never, former, current), education (< 12 years, ≥12 years), alcohol drinking status (never, former, current), BMI (< 30 kg/m^2^, ≥ 30 kg/m^2^), location (Arizona, Oklahoma, North and South Dakota), and total arsenic (log µg/g). Residual kurtosis was –0.14 for %iAs, %MMA, and %DMA. The number of pair relationships among the 487 participants was distributed as follows: 33 parent–offspring; 268 siblings; 93 avuncular; 36 half siblings; 2 double first cousins; 3 grand avuncular; 19 half avuncular; 87 first cousins; 9 half first cousins and half second cousins; 8 first cousins once removed (1 rem); 5 half first cousins; 6 half first cousins, 1 rem, and half second cousins, 1 rem.

*Linkage scan*. We found suggestive evidence in favor of QTLs for %iAs on chromosomes 5 (LOD = 2.03 at 39 cM, marker D5S416), 9 (LOD = 2.05 at 51 cM, between markers D9S171 and D9S161), and 11 (LOD = 1.91 at 9 cM, marker D11S1338); and for %MMA on chromosome 9 (LOD = 2.10 at 162 cM, marker D9S158) [Figure 1 and see also Supplemental Material, Figure S2 (http://dx.doi.org/10.1289/ehp.1205305)]. The correction constants for computing empirical LOD scores were 0.99 for %iAs, 0.87 for %MMA, and 0.92 for %DMA. Thus, the adjusted LOD scores for %iAs remained very similar to the original scores (2.01, 2.03, and 1.89 for %iAs on chromosomes 5, 9, and 11, respectively), whereas the adjusted LOD for %MMA on chromosome 9 decreased to a greater extent (adjusted LOD = 1.83). A peak for %DMA on chromosome 10 within 2 Mb (megabases) of *AS3MT* did not reach the level of suggestive evidence in favor of QTLs (LOD = 1.80 at 125 cM, between markers D10S192 and D10S597) (Figure 1; see also Supplemental Material, Figure S2).

In analyses stratified by geographic region, the peak in chromosome 5, the two peaks in chromosome 9, and the peak in chromosome 10 were smaller but present in all three regions [see Supplemental Material, Figure S2 (http://dx.doi.org/10.1289/ehp.1205305)]. There were also differences by region. In Oklahoma, there was suggestive evidence in favor of an additional QTL for %MMA on chromosome 18 (LOD = 2.21 at 22 cM, markers D18S63 and D18S452) and for %DMA on chromosome 19 (LOD = 2.29 at 79 cM, marker D19S902). In North and South Dakota there were suggestive peaks for %MMA and %DMA on chromosome 12 (LOD = 1.89 at 56 cM and 2.93 at 61 cM, respectively, markers D12S345 and D12S85) and for %DMA on chromosome 16 (LOD = 1.95, at 39 cM, marker D16S3103).

## Discussion

This population-based study in American Indian communities from Arizona, Oklahoma, and North and South Dakota supports a genetic contribution to variability in the distribution of urine arsenic metabolites. The heritability of urine arsenic metabolites ranged between 50% for %MMA and 59% for %DMA, with no major differences by study region. Based on a QTL linkage scan in a subset of our study population, we identified several genetic loci that may contribute to the pattern of urine arsenic metabolites. The presence of multiple loci indicates that, as suspected, multiple genes may be involved. Our unbiased discovery approach identified potential areas of the genome that may be related to metabolic processes and excretion of arsenic species in urine. Several biologically plausible genes (Hernandez and Marcos 2008) were located in those areas, including three histone methyltransferases [PR domain zinc finger protein 9 (*PRDM9*) on chromosome 5, histone methyltransferase *EHMT1* on chromosome 9, and ribosome RNA-processing protein 8 (*RRP8*) on chromosome 11] and one aquaglyceroporin, aquaglyceropin 3 (*AQP3*) on chromosome 9. The peak for %DMA on chromosome 10 was within 2 Mb of *AS3MT* but did not reach the level of suggestive evidence. Larger linkage studies and fine mapping are needed to confirm the relevance of these findings and to identify the genes and variants related to arsenic methylation patterns in urine.

In human populations, arsenic metabolism is commonly studied by measuring the relative proportion of inorganic and methylated arsenic metabolites in urine (Vahter 2000). Determinants of arsenic metabolism include sex, smoking, alcohol intake, nutritional status, BMI, and race/ethnicity (Gamble et al. 2005; Gomez-Rubio et al. 2011, 2012; Hopenhayn-Rich et al. 1996a; Hsueh et al. 2003; Navas-Acien et al. 2009; Steinmaus et al. 2005). Men, smokers, people who drink alcohol, and people with nutritional deficiencies have higher %MMA and lower %DMA in urine (Gamble et al. 2005; Hsueh et al. 2003; Navas-Acien et al. 2009). Nutritional deficiencies, especially low folate and selenium levels, have been associated with lower arsenic methylation capacity and could play an important role in arsenic toxicity (Christian et al. 2006; Gamble et al. 2006; Heck et al. 2007; Hsueh et al. 2003). On the other hand, obesity and indigenous American ancestry have been associated with increased %DMA in urine (Gomez-Rubio et al. 2011, 2012). Studies on the role of arsenic exposure levels in arsenic metabolism have been inconsistent. Some studies have shown no relationship between exposure levels and methylation patterns (Hopenhayn-Rich et al. 1996a, 1996b). Others have found increasing arsenic levels in drinking water were associated with higher %MMA and lower %DMA in urine (Heck et al. 2007; Lindberg et al. 2008). In our study, at low-to-moderate arsenic exposure levels, the distribution of arsenic species was similar for participants with urine total arsenic concentrations below and above the median. Nongenetic factors could result in false positives in a linkage study if they mimic Mendelian patterns, an unlikely occurrence. Regarding indigenous American ancestry, our findings are consistent with those from Andean (Engstrom et al. 2010; Hopenhayn-Rich et al. 1996b; Vahter et al. 1995) and Northwest Mexican (Gomez-Rubio et al. 2012) communities characterized by higher %DMA in urine compared with populations in Europe and Asia (Chiou et al. 1997; Gamble et al. 2005; Kile et al. 2011; Lindberg et al. 2007). The different arsenic methylation profile in American Indian populations could be partly genetically determined.

A number of studies have estimated associations of genetic polymorphisms with arsenic metabolism in different populations (Chiou et al. 1997; Engstrom et al. 2011; Gomez-Rubio et al. 2010; Hernandez and Marcos 2008; Hernandez et al. 2008; Lindberg et al. 2007; McCarty et al. 2007; Meza et al. 2005; Schlawicke et al. 2007, 2009; Vahter 2000), as measured by the relative distribution of inorganic and methylated metabolites in urine. So far, those studies have evaluated only a limited number of polymorphisms, and no genome-wide scans, using either SNPs or microsatellite markers (STR), are available. Polymorphisms in *AS3MT* have been associated with urine arsenic metabolites in populations in Argentina (Engstrom et al. 2011; Schlawicke et al. 2007, 2009), Chile (Hernandez et al. 2008), Mexico (Gomez-Rubio et al. 2010; Meza et al. 2005), Bangladesh (Engstrom et al. 2011), and Central Europe (Lindberg et al. 2007). Moreover, the functional relevance of *AS3MT* is well established (Chen et al. 2011; Drobna et al. 2006; Thomas et al. 2004; Wood et al. 2006). In our study, the peak on chromosome 10 close to *AS3MT* did not reach the level of suggestive evidence for linkage, and larger linkage studies within the overall SHFS population, as well as association studies, are needed to further evaluate the relevance of *AS3MT* in our population. Recently, another methyltransferase, N-6 adenine-specific DNA methyltransferase (*N6AMT1*), was shown *in vitro* to methylate arsenic in human urothelial cells (Ren et al. 2011). However, there was no evidence in our linkage scan of a peak in the vicinity of this gene, which is located on chromosome 21. Some of the peaks in our study, however, were within 2 Mb of histone methyltransferases (*PRDM9*, *EHMT1* and *RRP8*). In Oklahoma, the peak for %MMA on chromosome 18 was close to RNA methyltransferase (*RNMT*); in North and South Dakota, the peaks for %MMA and %DMA on chromosome 12 were close to methyltransferase-like 20 (*METTL20*). The possible role of these methylatransferases in arsenic metabolism has not been previously evaluated. The localization of QTLs in gene regions encompassing several histone methyltransferases is interesting given the increasingly recognized connections between arsenic and epigenetic modifications including histone methylation and acetylation (Arita and Costa, 2009; Baccarelli and Bollati, 2009; Hou et al. 2012; Martinez-Zamudio and Ha 2011).

In addition to methyltransferases, other genes have been suggested to play a role in arsenic metabolism, including glutathione and glutathione transferase genes, one-carbon metabolism and reduction genes, purine nucleoside phosphorylase genes, and transporter genes (Hernandez and Marcos 2008). Transporter genes could be important for the absorption and excretion of inorganic arsenic species (Hernandez and Marcos 2008). In our study, we found a peak for %iAs within 2 Mb of *AQP3* on chromosome 9. *In vitro* studies have shown that uptake of inorganic trivalent arsenicals is facilitated by several AQP cell membrane proteins (Calatayud et al. 2012; Liu et al. 2002, 2004). *In vivo*, *AQP9*-null mice had reduced arsenic clearance (Carbrey et al. 2009). In humans, polymorphisms in *AQP3* have recently been related to increased bladder cancer risk in individuals from New Hampshire who were exposed to arsenic in drinking water (Lesseur et al. 2012). Human studies are needed to evaluate the association of aquaglyceroporin polymorphisms with urine arsenic metabolites.

Our study has a number of limitations. First, the sample size for the linkage scan was small, particularly when stratified by region. Although the results of the heritability analyses were similar across the three regions, a larger study sample would be needed to confirm the consistency of the QTL linkage scan across regions and to evaluate gene × environment interactions by other characteristics such as arsenic exposure and sex. Ongoing arsenic speciation analysis in all SHFS participants and additional genetic analysis of a panel of polymorphisms in candidate genes potentially related to arsenic metabolism, including those informed by the present study, will allow us to determine if the linkages observed remain in the larger population as well as to study potential gene × environment interactions for arsenic related health effects.

A second limitation is that only one measurement of urine arsenic was available, although we confirmed the relative constancy of urine arsenic methylation patterns over a 10-year period in a pilot study of the same population (Navas-Acien et al. 2009). In addition, we could not determine if the loci associated with arsenic methylation patterns in urine were also associated with methylation patterns in other tissues or fluids, for instance, blood. Also, as in other linkage scans, peaks included relatively large genomic regions and substantial uncertainty remains regarding the specific genes involved. Finally, our population was exposed to low-to-moderate arsenic levels (from < 10 to > 50 µg/L in drinking water) and the relevance of the findings at different exposure levels is unknown. Strengths of the study include the availability of highly informative complex pedigrees for evaluating genetic determinants, the large sample size for the heritability analysis, and the high-quality standardized protocols used to recruit participants, conduct interviews and physical examinations, collect biological specimens, and perform laboratory analyses to measure arsenic species using highly sensitive methods (Lee et al. 1990; Scheer et al. 2012).

In conclusion, our heritability analysis and preliminary genome-wide linkage scan supports the hypothesis that genetic variation across the genome contributes substantially to the variability of urine arsenic methylation patterns in urine. The discovery and characterization of genes involved in arsenic metabolism is an important area of research as arsenic methylation patterns in urine have been related to cancer and cardiovascular disease risk (Chen et al. 2003; Del Razo et al. 1997; Hsueh et al. 1997; Kile et al. 2011; Steinmaus et al. 2006; Wu et al. 2006; Yu et al. 2000). Additional epidemiologic and experimental studies are needed to identify specific variants that are related to arsenic metabolism, confirm the findings in different populations, evaluate the function of potentially novel candidate genes such as histone methyltransferases and aquaglyceroporins, and evaluate the role of arsenic metabolism genes and of arsenic-gene interactions in arsenic-related toxicity and health effects.

## Supplemental Material

(836 KB) PDF
