# Supplementary material for: Heritability and Preliminary Genome-Wide Linkage Analysis of Arsenic Metabolites in Urine
Source: Environ Health Perspect. 2013 Jan 15;121(3):345–51. doi: 10.1289/ehp.1205305 (PMC3621197; doi:10.1289/ehp.1205305)
Supplement: (836 KB) PDF [file ehp.1205305.s001.pdf]

**SUPPLEMENTAL MATERIAL**

# Heritability and Preliminary Genome-Wide Linkage Analysis of Arsenic Metabolites in Urine

**Authors:** Maria Tellez-Plaza, Matthew O. Gribble, V. Saroja Voruganti, Kevin A. Francesconi, Walter Goessler, Jason G. Umans, Ellen K. Silbergeld, Eliseo Guallar, Nora Franceschini, Kari E. North, Wen. H. Kao, Jean W. MacCluer, Shelley A. Cole and Ana Navas-Acien

**TABLE OF CONTENTS**

**STATISTICAL METHODS, VARIANCE COMPONENT MODELS FOR GENERAL PEDIGREES CONDUCTED BY SOLAR. ..... 2**

*HERITABILITY MODEL* ..... 2

*QUANTITATIVE TRAIT LOCUS MODEL* ..... 3

**REFERENCES ..... 4**

**TABLE S1. PARTICIPANT CHARACTERISTICS ..... 5**

**TABLE S2. URINE ARSENIC METABOLITES BY PARTICIPANT CHARACTERISTICS ..... 6**

**FIGURE S1. CORRELATION MATRIX ..... 7**

**FIGURE S2. LINKAGE SCAN OF GENETIC LOCI ..... 8**

**TABLE S3. LIST OF SHORT TANDEM REPEAT (STR) MARKERS ..... 9**

**Statistical Methods, Variance component models for general pedigrees conducted by SOLAR.**

***Heritability model***

We estimated the heritability of urine arsenic metabolites (%iAs, %MMA and %DMA) by using a general pedigree variance-component method as implemented in the software Sequential Oligogenic Linkage Analysis Routines (SOLAR) (Blangero et al. 2013). SOLAR incorporates the information contained in the participants' pedigrees to obtain maximum likelihood estimates for the proportion of unexplained variance due to additive genetic effects from polygenes and the proportion of variance due to unmeasured environmental covariates, measurement error and non-additive genetic effects. The resultant polygenic model is specified as follows:

$$y_i = \mu + \Sigma^j_1 \beta_j \, x_{ij} + g_i + e_i \text{ [Equation 1]}$$

where $y_i$ is the observed urine arsenic metabolite (%iAs, %MMA or %DMA) for individual i; $\mu$ is the mean when all the covariates in the models are zero; $\beta_j$ is the vector of regression coefficients; $x_{ij}$ is the value of the covariable j in subject i; and $g_i$ and $e_i$ are the deviations from $\mu$ in the individual i which are attributable to additive genetic effects ($g_i$) and other sources of error ($e_i$) including unmeasured environmental effects, gene-gene interaction and gene-environment interaction. We assume that $g_i$ and $e_i$ are uncorrelated and normally distributed with mean 0 and variances $\sigma^2_g$ and $\sigma^2_{e.}$

To enable the analysis of arbitrary pedigree structures, the variance in the model is structured as the following covariance matrix:

$$\Omega = 2\,\Phi\,\sigma^2_g + I\,\sigma^2_e \text{ [Equation 2]}$$

where $\Phi$ is the matrix containing the kinship coefficients for all pairs of relatives in the data and $I$ is an identity matrix. Subsequently, the expected mean and covariance matrix for each pedigree are defined, and the likelihood of a pedigree is evaluated using the multivariate normal distribution and summing over all pedigrees. The heritability ($h^2$) is defined as the proportion of unexplained variance in the observed distribution of urine arsenic metabolite that is attributable

to additive genetic effects, or $h^2 = \sigma^2_g / (\sigma^2_g + \sigma^2_e)$. The p-values for $h^2$ are computed from a likelihood ratio test comparing the likelihood of the model in which the $\sigma^2_g$ is estimated to a model where $\sigma^2_g$ is constrained to be 0. These models are more deeply discussed by Hopper JL and Lange K (Hopper and Mathews 1982; Lange and Boehnke 1983).

***Quantitative trait locus model***

The linkage scan was based on variance component methods as implemented by SOLAR (Almasy and Blangero 1998). The model builds on the model in Equation 1 (see above) adding a term for *k* quantitative trait loci (QTLs), potentially associated to urine arsenic metabolites variability, where $q_{ik}$ is the normally distributed error term due to the k$^{th}$ QTL in individual i. The resulting equations for the observed urine arsenic metabolite values including the QTLs and corresponding variance are specified as follows:

$$y_i = \mu + \Sigma^j{}_1\beta_j\, x_{ij} + \Sigma^k{}_1 q_{ki} + g_i + e_i \text{ [Equation 3], and}$$

$$\Omega = \Sigma^k{}_1\Pi_k\sigma_{qk} + 2\,\Phi\,\sigma^2_g + I\,\sigma^2_e \text{ [Equation 4],}$$

where $\Pi_k$ is the identity-by-descent (IBD) matrix whose elements provide the probability of sharing genes identical by descent for a given pair of individuals at a given genetic marker locus potentially linked to a QTL, Φ is the kinship matrix and I is the identity matrix, $\sigma_{qk\_}$ refers to genetic variance due to the QTL and $\sigma^2_{g\_}$ refers to the residual additive genetic variance. Almasy L. and Blangero have described the variance component model for QTL linkage analysis in general pedigrees in more detail (Almasy and Blangero 1998; Blangero and Almasy 1997).

**References**


Almasy L, Blangero J. 1998. Multipoint quantitative-trait linkage analysis in general pedigrees. Am J Hum Genet May 62(5):1198-1211.

Blangero J, Almasy L. 1997. Multipoint oligogenic linkage analysis of quantitative traits. Genet Epidemiol 14(6):959-964.

Blangero J, Lange K, Almasy L, et al. SOLAR: Sequential Oligogenic Linkage Analysis Routines. 1999-2004; Available at: http://www.txbiomed.org/departments/genetics/genetics-detail?p=37. [Accessed January 7, 2013]

Hopper JL, Mathews JD. 1982. Extensions to multivariate normal models for pedigree analysis. Ann Hum Genet 46(Pt 4):373-383.

Lange K, Boehnke M. 1983. Extensions to pedigree analysis. IV. Covariance components models for multivariate traits. Am J Med Genet. 14(3):513-524.

**Supplemental Material, Table S1. Participant characteristics**

Table S1. Characteristics of Strong Heart Study participants with at least one relative within the cohort and STR markers measured (N=487)[a]

| | Arizona (N= 178) | Oklahoma (N= 184) | Dakotas (N= 125) | Overall (N= 487) |
|---|---|---|---|---|
| Age (years) | 54.3 (0.5) | 55.6 (0.5) | 55.2 (0.6) | 55.0 (0.3) |
| Sex (% males) | 29.2 (3.4) | 39.7 (3.6) | 36.0 (4.3) | 34.9 (2.2) |
| Education (%<High School) | 62.4 (3.6) | 29.9 (3.4) | 30.4 (4.1) | 41.9 (2.2) |
| BMI (kg/m$^2$) | 32.7 (0.4) | 31.0 (0.4) | 30.7 (0.5) | 31.6 (0.3) |
| Smoking status (%) | | | | |
| Former | 33.7 (3.6) | 33.7 (3.5) | 34.4 (4.3) | 33.9 (2.1) |
| Current | 16.9 (2.8) | 34.8 (3.5) | 40.8 (4.4) | 29.8 (2.1) |
| Current alcohol drinkers (%) | | | | |
| Former | 40.4 (3.7) | 41.8 (3.6) | 35.2 (4.3) | 39.6 (2.2) |
| Current | 41.0 (3.7) | 35.3 (3.5) | 52.8 (4.5) | 41.9 (2.2) |
| Total arsenic (µg/g)[b] | 19.22 (12.70, 26.93) | 7.93 (6.14, 11.46) | 8.73 (5.62, 15.36) | 11.19 (7.03, 18.86) |
| Arsenobetaine (µg/g) [b] | 0.72 (0.47, 1.25) | 0.70 (0.47, 1.20) | 0.54 (0.37, 1.28) | 0.66 (0.45, 1.27) |

[a]Percentages (standard errors) for categorical variables or means (standard errors) for continuous variables.

[b]Median (Interquartile range).

Total arsenic was measured directly using inductively coupled plasma mass spectrometry (ICPMS, see methods section).

**Supplemental Material, Table S2. Urine arsenic metabolites by participant characteristics**

Table S2. Median (interquartile range) of percentage urine arsenic species in the Strong Heart Study participants with at least one relative within the cohort

| | N | % Inorganic As | % MMA | % DMA |
|---|---|---|---|---|
| Overall | 487 | 7.6 (5.4, 10.3) | 13.7 (10.6, 16.9) | 78.8 (72.1, 83.1) |
| | | | | |
| Age, years | | | | |
| ≤55 | 271 | 8.2 (5.7, 10.4) | 13.4 (10.5, 17.1) | 78.3 (71.9, 82.5) |
| >55 | 216 | 6.9 (4.8, 10.3) | 13.9 (10.8, 16.8) | 79.6 (72.7, 83.8) |
| | | | | |
| Sex | | | | |
| Men | 170 | 8.8 (6.2, 12.3) | 15.4 (11.7, 18.4) | 75.4 (69.7, 81.1) |
| Women | 317 | 7.1 (4.8, 9.4) | 12.8 (10.1, 15.9) | 79.9 (74.8, 84.1) |
| | | | | |
| Study region | | | | |
| Arizona | 178 | 8.7 (5.9, 11.7) | 12.7 (10.3, 15.6) | 78.9 (72.1, 83.1) |
| Oklahoma | 184 | 7.0 (4.8, 9.3) | 13.3 (10.2, 16.2) | 79.2 (73.9, 84.2) |
| Dakotas | 125 | 7.3 (5.3, 10.3) | 15.9 (12.4, 18.5) | 77.2 (70.3, 81.1) |
| | | | | |
| Education | | | | |
| > 12 years | 283 | 7.2 (5.4, 9.8) | 13.7 (10.7, 17.1) | 78.8 (72.2, 83.3) |
| ≤ 12 years | 204 | 8.3 (5.5, 10.9) | 13.6 (10.5, 16.7) | 78.6 (72.1, 82.9) |
| | | | | |
| BMI, kg/m$^2$ | | | | |
| <30 | 201 | 8.7 (6.0, 11.8) | 15.5 (12.2, 18.4) | 76.1 (69.8, 81.1) |
| ≥ 30 | 286 | 7.1 (5.1, 9.4) | 12.4 (10.0, 15.5) | 80.4 (74.9, 84.3) |
| | | | | |
| Smoking | | | | |
| Never | 177 | 6.8 (5.2, 9.1) | 12.2 (9.7, 15.6) | 80.9 (75.6, 84.2) |
| Former | 165 | 7.4 (5.0, 10.1) | 13.7 (10.5, 16.8) | 78.8 (72.0, 83.7) |
| Current | 145 | 8.9 (6.1, 12.1) | 15.1 (12.4, 18.1) | 76.0 (70.4, 80.5) |
| | | | | |
| Alcohol drinking | | | | |
| Never | 90 | 6.6 (4.9, 8.6) | 12.2 (9.4, 15.9) | 80.8 (75.8, 85.7) |
| Former | 193 | 7.9 (5.5, 11.2) | 13.9 (11.1, 16.9) | 78.4 (71.7, 82.1) |
| Current | 204 | 8.0 (5.4, 10.5) | 14.2 (10.8, 17.3) | 78.2 (71.9, 82.7) |
| | | | | |
| Total Arsenic, µg/g | | | | |
| < 12.9 | 279 | 7.3 (5.4, 9.5) | 14.6 (11.2, 17.6) | 78.4 (71.9, 82.2) |
| ≥ 12.9 | 208 | 8.3 (5.4, 11.7) | 12.4 (9.9, 15.6) | 79.3 (72.4, 83.9) |
| | | | | |
| Arsenobetaine, µg/g | | | | |
| < 0.7 | 258 | 8.1 (5.8, 10.7) | 14.4 (11.2, 17.7) | 77.4 (71.8, 81.6) |
| ≥ 0.7 | 229 | 7.1 (4.7, 10.2) | 12.7 (10.0, 16.4) | 80.4 (73.7, 84.4) |

For BMI we selected 30 kg/m$^2$, a cut-off commonly used to classify individuals as obese and non-obese. For education we selected 12 years of education, as at least 12 years of education is consistent with the completion of high school. Total arsenic was measured directly using inductively coupled plasma mass spectrometry (ICPMS, see methods section).

**Supplemental Material, Figure S1. Correlation matrix**

Figure S1. Distribution and relationship of total arsenic concentrations (µg/g creatinine) and arsenic metabolites (%iAs, %MMA and %DMA) in urine (n=2,907).

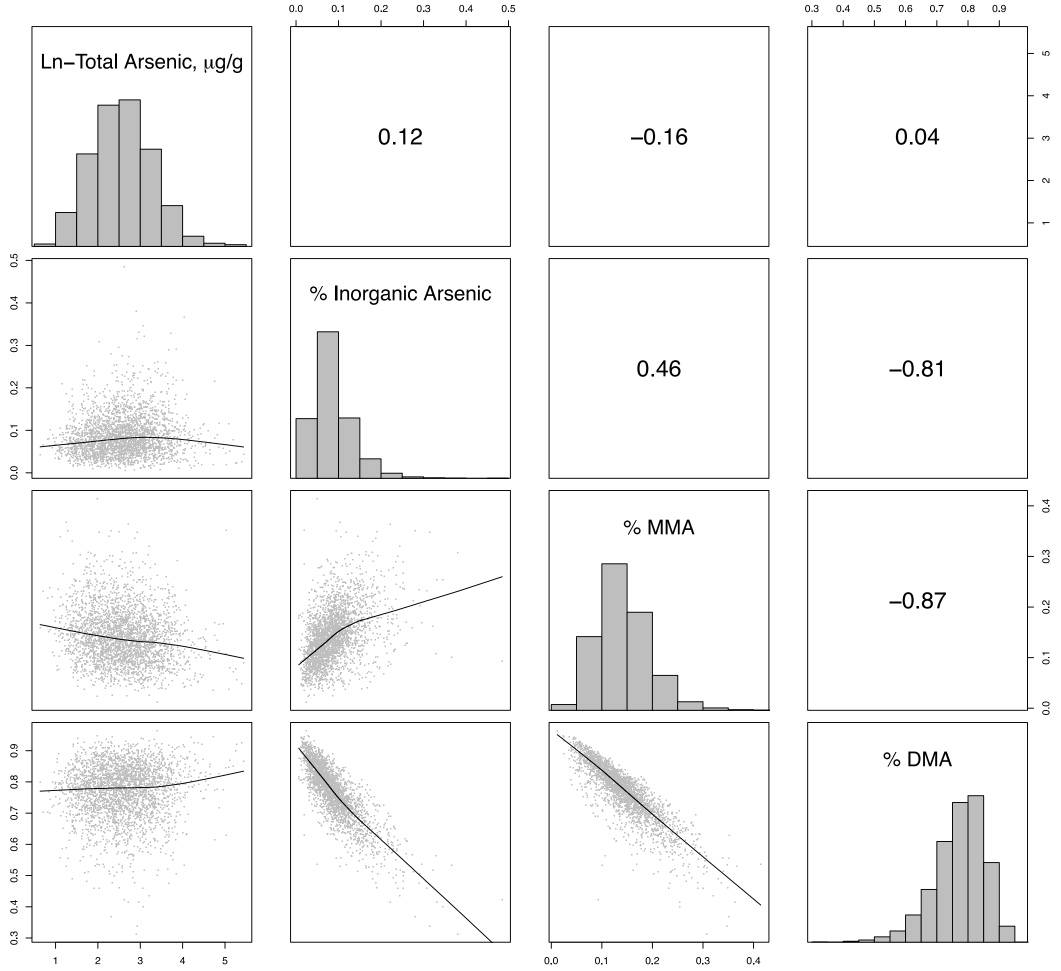


The diagonal shows the distribution of the variables in the correlation matrix. The upper diagonal panel shows the Spearman correlation coefficients for the corresponding variables in the correlation matrix. The lower diagonal panel shows the scatterplots and the smoothed relationship for the corresponding variables in the correlation matrix using the lowess command in R software (R-Development Core Team 2012, available at: http://cran.r-project.org/).

**Supplemental Material, Figure S2. Linkage scan of genetic loci** associated with urine arsenic metabolites in Strong Heart Study participants with short tandem repeat markers genotyped, stratified by study region

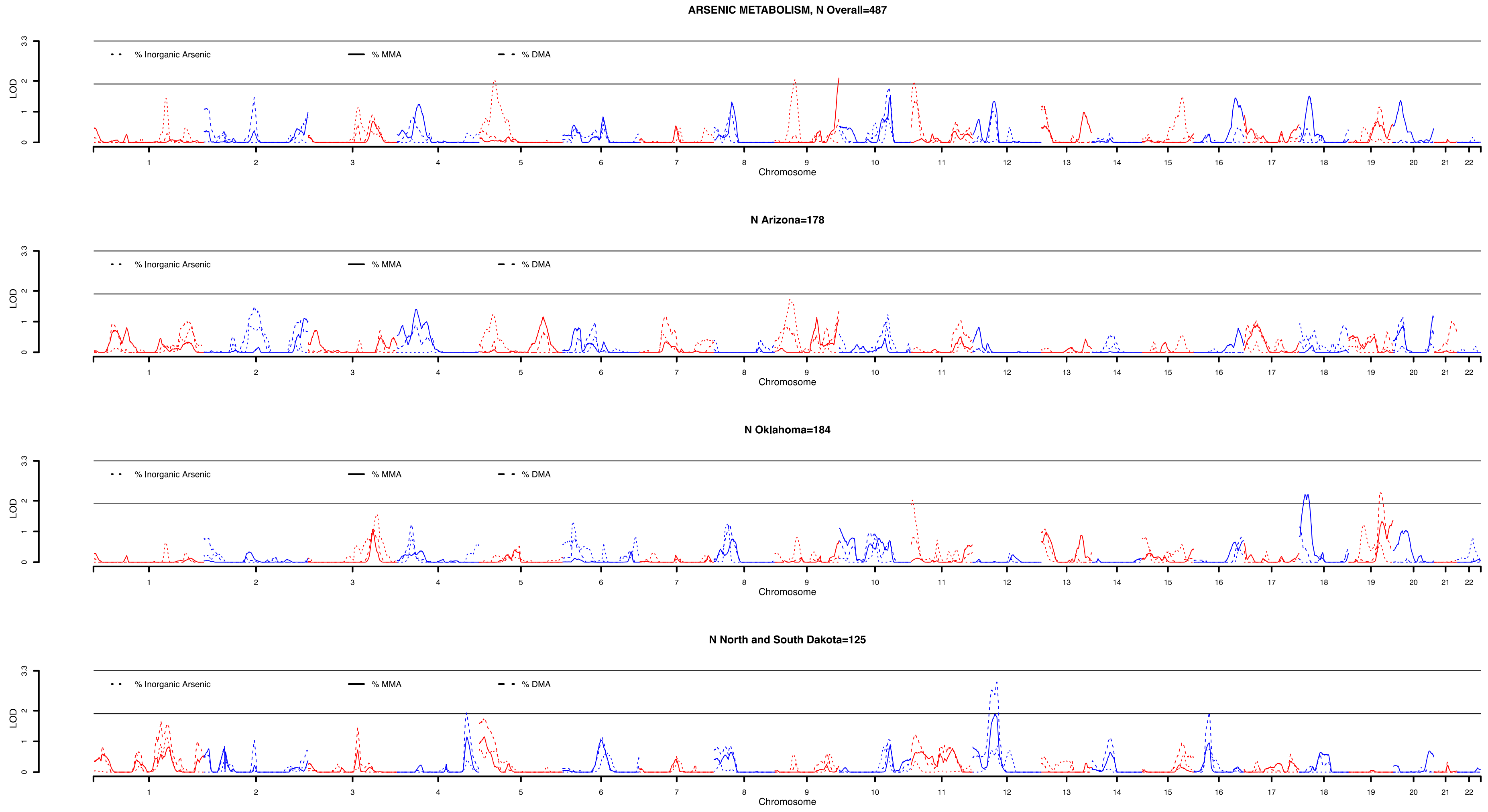


Models adjusted for age, age$^2$, sex, age*sex, age2*sex, smoking status (never, former, current), education (> 12 years, ≤ 12 years), body mass index (<30 and ≥30 kg/m$^2$), alcohol status (never, former, current), region (Dakotas, Oklahoma and Arizona) and total arsenic (log μg/g). Residual kurtosis was -0.05 for % inorganic arsenic, -0.05 for %MMA and -0.04 for %DMA. The horizontal lines represent LOD scores of 1.9 and 3.3, which are considered as suggestive and confirmed evidence in favor of linkage with a causal locus (Lander E and Kruglyak L. Nat Genet 1995;11:241-247).

**Supplemental Material, Table S3. List of short tandem repeat (STR) markers used in the Strong Heart Family Study.** The chromosomal location is based on Haldane centimorgans.

| Chromosome | Marker | Location |
|---|---|---|
| 1 | D1S468 | 4.16 |
| | D1S214 | 12.35 |
| | D1S450 | 16.95 |
| | D1S2667 | 20.32 |
| | D1S2697 | 29.88 |
| | D1S199 | 38.34 |
| | D1S234 | 45.44 |
| | D1S255 | 59.79 |
| | D1S2797 | 70.2 |
| | D1S2890 | 82.35 |
| | D1S230 | 89.57 |
| | D1S2841 | 105.49 |
| | D1S207 | 108.87 |
| | D1S2868 | 118.5 |
| | D1S206 | 124.55 |
| | D1S2726 | 134.14 |
| | D1S252 | 141.25 |
| | D1S498 | 147.13 |
| | D1S484 | 159.84 |
| | D1S2878 | 168.29 |
| | D1S196 | 171.94 |
| | D1S218 | 178.92 |
| | D1S238 | 191.32 |
| | D1S413 | 197.84 |
| | D1S249 | 210.08 |
| | D1S425 | 218.18 |
| | D1S213 | 228.76 |
| | D1S2800 | 244.37 |
| | D1S2785 | 261.73 |
| | D1S2842 | 266.17 |
| | D1S2836 | 276.53 |
| | | |
| 2 | D2S319 | 12.31 |
| | D2S2211 | 18.3 |
| | D2S162 | 23.43 |
| | D2S168 | 29.74 |
| | D2S305 | 42.37 |
| | D2S165 | 52.05 |
| | D2S367 | 59.1 |

| | | |
|---|---|---|
| | D2S2259 | 68.38 |
| | D2S391 | 73.72 |
| | D2S337 | 85.62 |
| | D2S2368 | 90.93 |
| | D2S286 | 101.9 |
| | D2S2333 | 110.84 |
| | D2S2216 | 113.54 |
| | D2S160 | 127.14 |
| | D2S347 | 137.61 |
| | D2S112 | 148.29 |
| | D2S151 | 160.24 |
| | D2S142 | 168.06 |
| | D2S2330 | 174.06 |
| | D2S335 | 181.11 |
| | D2S364 | 191.09 |
| | D2S117 | 198.11 |
| | D2S325 | 208.47 |
| | D2S2382 | 217.52 |
| | D2S126 | 227.19 |
| | D2S396 | 239.32 |
| | D2S206 | 244.07 |
| | D2S338 | 252.65 |
| | D2S125 | 262.74 |
| | | |
| 3 | D3S1297 | 4.95 |
| | D3S1304 | 20.31 |
| | D3S1263 | 30.53 |
| | D3S3659 | 45.15 |
| | D3S1266 | 51.92 |
| | D3S1277 | 61.67 |
| | D3S1289 | 75.46 |
| | D3S1300 | 84.03 |
| | D3S1285 | 91.18 |
| | D3S1566 | 96.72 |
| | D3S3681 | 109.69 |
| | D3S1271 | 114.57 |
| | D3S1278 | 125.11 |
| | D3S1267 | 132.82 |
| | D3S1292 | 141.43 |
| | D3S1569 | 153.31 |
| | D3S1279 | 163.02 |
| | D3S1614 | 172.88 |
| | D3S1565 | 181 |

| | | |
|---|---|---|
| | D3S1262 | 197.62 |
| | D3S1580 | 205.46 |
| | D3S1601 | 211.65 |
| | D3S1311 | 223.98 |
| | | |
| 4 | D4S412 | 4.52 |
| | D4S2935 | 13.24 |
| | D4S403 | 27.62 |
| | D4S419 | 36.07 |
| | D4S391 | 48.62 |
| | D4S405 | 61.72 |
| | D4S1592 | 74.36 |
| | D4S392 | 82.1 |
| | D4S2964 | 90.26 |
| | D4S1534 | 95.04 |
| | D4S1572 | 109.5 |
| | D4S406 | 116.82 |
| | D4S402 | 124.26 |
| | D4S1575 | 133.91 |
| | D4S424 | 141.27 |
| | D4S413 | 154.83 |
| | D4S1597 | 166.41 |
| | D4S1539 | 172.85 |
| | D4S415 | 176.12 |
| | D4S1535 | 192.54 |
| | D4S426 | 206.46 |
| | | |
| 5 | D5S1981 | 1.2 |
| | D5S406 | 12.98 |
| | D5S630 | 24.73 |
| | D5S416 | 37.82 |
| | D5S419 | 47.48 |
| | D5S426 | 58.25 |
| | D5S418 | 66.17 |
| | D5S407 | 73.57 |
| | D5S647 | 80.58 |
| | D5S424 | 92.74 |
| | D5S641 | 101.1 |
| | D5S428 | 103.9 |
| | D5S644 | 110.69 |
| | D5S433 | 114.77 |
| | D5S2027 | 120.17 |
| | D5S471 | 127.38 |

| | | |
|---|---|---|
| | D5S2115 | 139.28 |
| | D5S436 | 150 |
| | D5S410 | 161.55 |
| | D5S422 | 169.32 |
| | D5S400 | 180.4 |
| | D5S408 | 209.3 |
| | | |
| 6 | D6S1574 | 15.95 |
| | D6S309 | 21.34 |
| | D6S470 | 24.65 |
| | D6S289 | 35.97 |
| | D6S422 | 44.27 |
| | D6S276 | 49.4 |
| | D6S1610 | 60.02 |
| | D6S257 | 81.56 |
| | D6S460 | 92.65 |
| | D6S462 | 100.04 |
| | D6S300 | 103.02 |
| | D6S287 | 122.15 |
| | D6S262 | 133.64 |
| | D6S292 | 139.36 |
| | D6S308 | 147.97 |
| | D6S441 | 163.69 |
| | D6S1581 | 173.43 |
| | D6S264 | 184.73 |
| | D6S281 | 192.35 |
| | D6S446 | 193.14 |
| | | |
| 7 | D7S531 | 7.67 |
| | D7S517 | 8.86 |
| | D7S513 | 23.1 |
| | D7S507 | 32.2 |
| | D7S493 | 37.65 |
| | D7S516 | 45.07 |
| | D7S484 | 55.58 |
| | D7S510 | 61.59 |
| | D7S519 | 69.87 |
| | D7S502 | 80.58 |
| | D7S669 | 90.83 |
| | D7S630 | 101.52 |
| | D7S657 | 105.09 |
| | D7S515 | 113.01 |
| | D7S486 | 124.27 |

| | | |
|---|---|---|
| | D7S530 | 132.61 |
| | D7S640 | 140.97 |
| | D7S684 | 149.07 |
| | D7S661 | 154.31 |
| | D7S636 | 165.35 |
| | D7S798 | 172.99 |
| | D7S2423 | 186.83 |
| | | |
| 8 | D8S264 | 3.47 |
| | D8S277 | 15.76 |
| | D8S550 | 21.88 |
| | D8S549 | 26.98 |
| | D8S258 | 35.88 |
| | D8S1771 | 44.92 |
| | D8S505 | 55.63 |
| | D8S285 | 69.83 |
| | D8S260 | 75.18 |
| | D8S270 | 99.18 |
| | D8S1784 | 114.59 |
| | D8S514 | 126.9 |
| | D8S284 | 142.34 |
| | D8S272 | 152.77 |
| | | |
| 9 | D9S288 | 8.53 |
| | D9S286 | 18.34 |
| | D9S285 | 34.59 |
| | D9S157 | 37.33 |
| | D9S171 | 46.84 |
| | D9S161 | 52.43 |
| | D9S1817 | 57.85 |
| | D9S273 | 68.12 |
| | D9S175 | 72.25 |
| | D9S167 | 82.73 |
| | D9S283 | 95 |
| | D9S287 | 100.82 |
| | D9S1690 | 106.26 |
| | D9S1677 | 115.15 |
| | D9S1776 | 124 |
| | D9S1682 | 131.25 |
| | D9S290 | 138.99 |
| | D9S164 | 149.72 |
| | D9S1826 | 161.18 |
| | D9S158 | 161.76 |

| | | |
|---|---|---|
| 10 | D10S249 | 1.19 |
| | D10S591 | 14.76 |
| | D10S189 | 20.36 |
| | D10S547 | 28.5 |
| | D10S1653 | 38.66 |
| | D10S548 | 44.41 |
| | D10S197 | 51.11 |
| | D10S208 | 61.08 |
| | D10S196 | 71.44 |
| | D10S1652 | 82.09 |
| | D10S537 | 90.78 |
| | D10S1686 | 106.69 |
| | D10S185 | 115.25 |
| | D10S192 | 122.74 |
| | D10S597 | 128.73 |
| | D10S1693 | 140.61 |
| | D10S587 | 151.27 |
| | D10S217 | 162.82 |
| | D10S1651 | 175.11 |
| | D10S212 | 180.5 |
| | | |
| 11 | D11S4046 | 0 |
| | D11S1338 | 9.97 |
| | D11S902 | 26.11 |
| | D11S904 | 44.32 |
| | D11S935 | 53.7 |
| | D11S905 | 58.2 |
| | D11S4191 | 65.85 |
| | D11S987 | 73.34 |
| | D11S1314 | 79.88 |
| | D11S937 | 84.91 |
| | D11S901 | 89.71 |
| | D11S4175 | 94.3 |
| | D11S898 | 105 |
| | D11S908 | 117.36 |
| | D11S925 | 125.75 |
| | D11S4151 | 134.91 |
| | D11S1320 | 149.32 |
| | D11S968 | 154.92 |
| | | |
| 12 | D12S352 | 0 |
| | D12S99 | 15.49 |

| | | |
|---|---|---|
| | D12S336 | 24.55 |
| | D12S364 | 31.91 |
| | D12S310 | 36.86 |
| | D12S1617 | 46.67 |
| | D12S345 | 56.05 |
| | D12S85 | 61.41 |
| | D12S368 | 67.52 |
| | D12S83 | 75.06 |
| | D12S326 | 92.48 |
| | D12S351 | 102.6 |
| | D12S346 | 112.78 |
| | D12S78 | 117.92 |
| | D12S79 | 133.95 |
| | D12S86 | 140.28 |
| | D12S324 | 151.13 |
| | D12S1659 | 162.27 |
| | D12S1723 | 172.4 |
| | | |
| 13 | D13S175 | 3.47 |
| | D13S217 | 22.94 |
| | D13S171 | 31.89 |
| | D13S218 | 40.33 |
| | D13S263 | 44.28 |
| | D13S153 | 52.87 |
| | D13S156 | 71.16 |
| | D13S170 | 77.67 |
| | D13S265 | 82.35 |
| | D13S159 | 93.61 |
| | D13S158 | 100.73 |
| | D13S173 | 111.41 |
| | D13S1265 | 116.67 |
| | D13S285 | 126.4 |
| | | |
| 14 | D14S261 | 4.52 |
| | D14S283 | 15.3 |
| | D14S275 | 22.95 |
| | D14S70 | 37.43 |
| | D14S288 | 45.16 |
| | D14S276 | 55.74 |
| | D14S63 | 64.95 |
| | D14S258 | 70.03 |
| | D14S74 | 79.76 |
| | D14S280 | 96.33 |

| | | |
|---|---|---|
| | D14S65 | 108.62 |
| | D14S985 | 118.3 |
| | D14S292 | 125.64 |
| | | |
| 15 | D15S128 | 6.06 |
| | D15S1002 | 15.51 |
| | D15S165 | 23.42 |
| | D15S1007 | 29.75 |
| | D15S1012 | 39.25 |
| | D15S994 | 43.89 |
| | D15S978 | 49.51 |
| | D15S117 | 57.9 |
| | D15S153 | 69.69 |
| | D15S131 | 78.27 |
| | D15S205 | 91.16 |
| | D15S127 | 99.44 |
| | D15S130 | 111.11 |
| | D15S120 | 129.9 |
| | | |
| 16 | D16S423 | 14.64 |
| | D16S404 | 25.69 |
| | D16S3075 | 30.38 |
| | D16S3103 | 39.13 |
| | D16S3046 | 44.2 |
| | D16S3068 | 50.81 |
| | D16S3136 | 61.8 |
| | D16S415 | 68.52 |
| | D16S503 | 83.55 |
| | D16S515 | 93.91 |
| | D16S516 | 100.45 |
| | D16S3091 | 111.96 |
| | D16S520 | 125.99 |
| | | |
| 17 | D17S849 | 0.63 |
| | D17S831 | 7.35 |
| | D17S938 | 17.45 |
| | D17S1852 | 31.26 |
| | D17S799 | 37.74 |
| | D17S921 | 42.34 |
| | D17S1857 | 47.4 |
| | D17S798 | 56.73 |
| | D17S1868 | 75.89 |
| | D17S787 | 82.77 |

| | | |
|---|---|---|
| | D17S944 | 94.6 |
| | D17S949 | 104.95 |
| | D17S785 | 117.48 |
| | D17S784 | 132.09 |
| | D17S928 | 138.44 |
| | | |
| 18 | D18S59 | 1.4 |
| | D18S63 | 9.74 |
| | D18S452 | 18.14 |
| | D18S464 | 33.23 |
| | D18S53 | 38.66 |
| | D18S478 | 52.14 |
| | D18S1102 | 59.5 |
| | D18S474 | 74.33 |
| | D18S64 | 83.8 |
| | D18S68 | 90.54 |
| | D18S61 | 99.56 |
| | D18S1161 | 110.48 |
| | D18S462 | 118.57 |
| | D18S70 | 123.94 |
| | | |
| 19 | D19S209 | 11.89 |
| | D19S216 | 17.38 |
| | D19S884 | 26.62 |
| | D19S221 | 33.51 |
| | D19S226 | 37.53 |
| | D19S414 | 55.22 |
| | D19S220 | 65.1 |
| | D19S420 | 70.51 |
| | D19S902 | 77.81 |
| | D19S888 | 96.51 |
| | D19S418 | 106.92 |
| | D19S210 | 111.68 |
| | | |
| 20 | D20S117 | 2.94 |
| | D20S889 | 12.15 |
| | D20S115 | 25.13 |
| | D20S186 | 35.24 |
| | D20S112 | 45.1 |
| | D20S195 | 57.3 |
| | D20S107 | 62.77 |
| | D20S119 | 70.48 |
| | D20S178 | 74.95 |

| | | |
|---|---|---|
| | D20S196 | 79.69 |
| | D20S100 | 90.29 |
| | D20S171 | 100.25 |
| | D20S173 | 101.32 |
| | | |
| 21 | D21S1256 | 13.2 |
| | D21S1899 | 16.08 |
| | D21S1914 | 24.9 |
| | D21S263 | 33.15 |
| | D21S1252 | 43.79 |
| | D21S266 | 57.06 |
| | | |
| 22 | D22S420 | 3.05 |
| | D22S539 | 15.63 |
| | D22S315 | 23.71 |
| | D22S280 | 38.53 |
| | D22S283 | 43.61 |
| | D22S423 | 50.75 |
| | D22S274 | 58.24 |
